# Evaluation of [^68^ Ga]Ga-PSMA-I&T PET/CT with additional late scans of the pelvis in prostate-specific antigen recurrence using the PROMISE criteria

**DOI:** 10.1186/s13550-022-00938-3

**Published:** 2022-10-09

**Authors:** Daniel Koehler, Markus Sauer, Amir Karimzadeh, Ivayla Apostolova, Susanne Klutmann, Gerhard Adam, Sophie Knipper, Tobias Maurer, Christoph Berliner

**Affiliations:** 1 Department of Diagnostic and Interventional Radiology and Nuclear Medicine, University Medical Center Hamburg-Eppendorf, Martinistraße 52, 20246 Hamburg, Germany; 2 Martini-Klinik Prostate Cancer Center, University Hospital Hamburg-Eppendorf, Hamburg, Germany; 3 Department of Urology, University Hospital Hamburg-Eppendorf, Hamburg, Germany; 4 Present Address: Department of Nuclear Medicine, University of Duisburg-Essen and German Cancer Consortium (DKTK)-University Hospital Essen, Essen, Germany

**Keywords:** Prostate cancer, PSA recurrence, PSMA I&T, Biphasic imaging

## Abstract

**Background:**

PSMA PET/CT is the recommended imaging test in cases with prostate-specific antigen (PSA) recurrence after primary therapy of prostate cancer (PCa). However, imaging protocols remain a topic of active research. The aim of the presented study was to examine the impact of additional late scans of the pelvis in [^68^ Ga]Ga-PSMA-I&T PET/CT of patients with rising PSA after prostatectomy.

**Methods:**

A total of 297 patients (median PSA 0.35 ng/ml, interquartile range (IQR) 0.2–0.8) who underwent early whole-body [^68^ Ga]Ga-PSMA-I&T PET/CT (median dose 141 MBq, IQR 120–163; median 86 min, IQR 56–107) and additional late scans of the pelvis (median 180 min, IQR 170–191) were investigated retrospectively. Early and late images were staged separately according to the PROMISE criteria and compared with a final consensus of both. Standardized uptake values were analyzed for early and late scans.

**Results:**

One hundred and thirty-four (45.1%) [^68^ Ga]Ga-PSMA-I&T PET/CT showed evidence of recurrent PCa (114/38.4% early, 131/44.1% late). Of 195 lesions, 144 (73.8%) were identified correctly on early scans. 191 (97.9%) lesions were detected on late imaging. The lesion SUVmax (median 3.4, IQR 0.4–6.5 vs. median 3.9, IQR 2.6–8.2) as well as the SUVmax to background ratio (median 9.4, IQR 1.7–19.1 vs. median 15.5, IQR 9.6–34.1) increased significantly between the imaging time points (*p* < 0.01, respectively). Compared to the final consensus, the miTNM-staging of early scans changed in 58 (19.5%) cases. Of these, 31 patients (10.4%) with negative early scans (T0 N0 M0) were upstaged. Twenty-seven (9.1%) patients with PCa characteristic lesions on early imaging (> T0 N0 M0) were up- and/or downstaged. In 4 (1.3%) cases, PCa-related lesions were only detectable on early PET/CT leading to upstagings of late imaging.

**Conclusions:**

Additional late scans of the pelvis in [^68^ Ga]Ga-PSMA-I&T PET/CT detected more lesions and an increasing contrast compared to early imaging. This influenced the final miTNM-staging substantially.

**Supplementary Information:**

The online version contains supplementary material available at 10.1186/s13550-022-00938-3.

## Background

Prostate cancer (PCa) is the most common cancer in men and the third most common cancer-related cause of death in the male population in Europe [1]. Rising prostate-specific antigen (PSA) values after definitive treatment occur frequently and represent an independent risk factor for cancer-specific mortality [2–5]. However, an increasing PSA after prostatectomy or radiation therapy alone is not sufficient to determine the risk of distant metastasis. To offer the most adequate treatment options in this patient group, it is necessary to differentiate local recurrence, locoregional lymph node metastases, and distant metastases [6, 7]. In this context, ^68^ Ga-labeled prostate-specific membrane antigen (PSMA) targeting PET/CT has demonstrated to localize PCa-related lesions with high sensitivity significantly impacting clinical decision making [8–13]. Moreover, it is the recommended diagnostic test in cases of biochemical recurrence if patients are fit for curative treatment [6]. While a guideline by the SNMMI and EANM that addresses imaging protocols of ^68^ Ga-labeled PSMA PET/CT already exists [14], the topic remains a field of active research. For example, early scans in the first 10 min after radioligand injection may help to visualize pelvic lesions with pathological PSMA expression before urinary activity interferes with image interpretation [15, 16]. On the other hand, several studies have described an increasing PSMA ligand uptake from standard to late imaging in most PCa-related lesions [17–22]. This was often paralleled by a decreasing tracer uptake of the background tissue [17, 21, 23]. The resulting improvement of the tumor-to-background ratio may lead to higher detection rates on late scans. But data on the matter of additional late imaging are still discussed controversially [17, 21, 23, 24], and results regarding biphasic PET acquisition of the theranostic compound PSMA-I&T are scarce [24].

The aim of this study was to evaluate the impact of additional late scans of the pelvis in [^68^ Ga]Ga-PSMA-I&T PET/CT using the PROMISE criteria [25]. For this, a highly selected patient collective of men with rising PSA values after radical prostatectomy without neoadjuvant or adjuvant therapy was investigated.

## Methods

### Study population

**Fig. 1:**
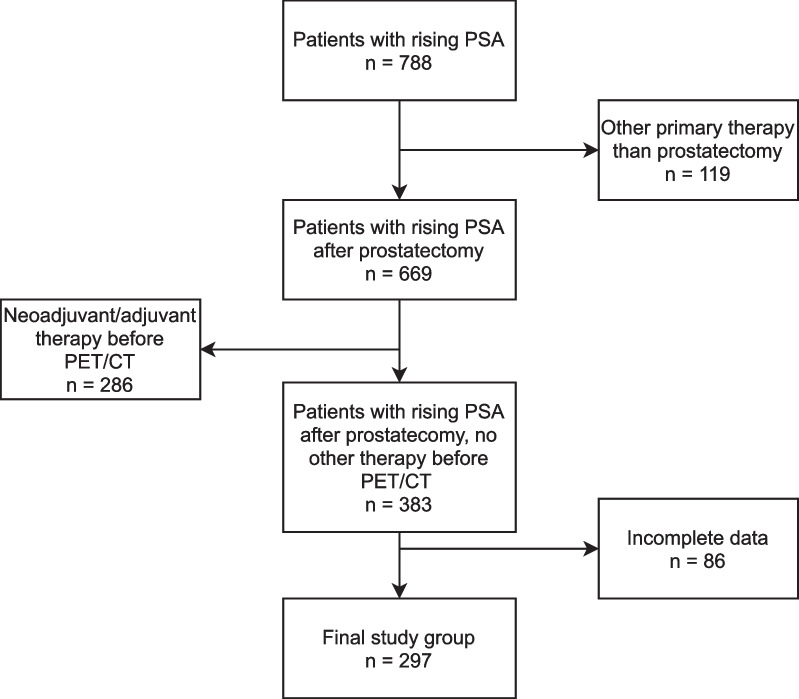
Case selection flowchart

Data of patients with rising PSA after primary therapy who were referred to our institution for [^68^ Ga]Ga-PSMA-I&T PET/CT between April 2016 and December 2019 were analyzed retrospectively. Cases were excluded if they had received any other treatment than radical prostatectomy before PET/CT (i.e., external beam radiation, brachytherapy, focal therapy, androgen deprivation therapy, chemotherapy) (Fig. 1). The final study cohort comprised 297 consecutive patients (median PSA 0.35, interquartile range (IQR) 0.2–0.8). Patient characteristics are summarized in Table 1.

**Table 1:** Clinical patient characteristics

Age (years)		Median 69, IQR 64—73
PSA at RP (ng/ml)		Median 8.1, IQR 5.6—12.6
pT-stage RP	T2	126 (42.4%)
	T3	170 (57.2%)
	T4	1 (0.3%)
ISUP grade	1	11 (3.7%)
	2	151 (50.8%)
	3	99 (33.3%)
	4	9 (3.0%)
	5	27 (9.1%)
Resection margin	R0	258 (86.9%)
	R1	39 (13.1%)
pN-stage RP	N0	224 (75.4%)
	N1	50 (16.8%)
	No LN dissected	23 (7.7%)
RP to PET (months)		Median 37, IQR 15–75
PSA at PET/CT (ng/ml)	< 0.2	45 (15.2%)
	0.2–0.5	148 (49.8%)
	> 0.5–1.0	41 (13.8%)
	> 1.0	63 (21.2%)

*IQR* interquartile range; *ISUP* international society of urological pathology; *LN* lymph node; *PSA* prostate-specific antigen; and *RP* radical prostatectomy

The local institutional review board (IRB) approved this retrospective single-center study and waived the requirement for informed consent (PV7316).

### Preparation of the PSMA ligand

[^68^ Ga]Ga-PSMA-I&T was produced and applicated according to §13 (2b) Arzneimittelgesetz (German drug law). It was synthesized using a GRP module (Scintomics GmbH, Fürstenfeldbruck, Germany) connected to a [^68^Ge]/[^68^ Ga] generator (50 mCi Gallia Pharma Generator, Eckert & Ziegler Radiopharma, Berlin, Germany) with a disposable cassette (ABX Scintomics, Radeberg, Germany). PSMA-I&T (Scintomics GmbH, Fürstenfeldbruck, Germany) was used for labeling. Labeling efficiency was determined by instant thin-layer chromatography and high-performance liquid chromatography. Gamma-count test, pH level testing, and endotoxin testing were performed as quality assurance measures.

### PET/CT imaging

A median dose of 141 MBq (IQR 120–163) [^68^ Ga]Ga-PSMA-I&T was injected intravenously. All patients were instructed to drink 1 L of water before PET acquisition. No diuretics were administered. Examinations were performed on a Gemini GXL10 scanner (*n* = 136, median PSA 0.35, IQR 0.2–0.8; Philips, Cleveland, Ohio, USA) or a Vereos scanner (*n* = 161, median PSA 0.35, IQR 0.2–0.7; Philips, Cleveland, Ohio, USA).

The median uptake time from injection to early whole-body scans was 86 min (IQR 56–107) and 180 min (IQR 170–191) to late scans of the pelvis. First, a low-dose non-enhanced CT (120 kV, mA modulated) was performed for attenuation correction. The following PET acquisition was conducted with 120 s per bed position for the head and thorax, 150 s for the abdomen, and 90 s for the lower extremities. Late scans of the pelvis were performed with 240 s per bed position. Transverse PET slices were reconstructed in a 144 × 144 matrix using an iterative three-dimensional line-of-response reconstruction algorithm (Gemini GXL 10) or a blob ordered-subsets time-of-flight reconstruction with two iterations and eleven subsets (Vereos). If no contraindications against the intravenous application of CT contrast medium (i.e., manifest hyperthyroidism, allergy) were present, a contrast-enhanced CT from the skull base to the mid-thigh was performed after early whole-body imaging. The scans were acquired in portal venous phase 100 s after the injection of 140 ml of Imeron 300 (Bracco Imaging, Konstanz, Germany) with 100–130 kV and an adaptive dose modulation (*n* = 285, 96%). The remaining patients (*n* = 12, 4%) only received a non-enhanced CT for attenuation correction.

### Image analysis and quantification of tracer uptake

All [^68^ Ga]Ga-PSMA-I&T PET/CT scans were analyzed by one board-certified nuclear medicine physician and one board-certified radiologist. Contrast-enhanced CT scans were used for morphological characterization of PSMA-positive lesions if available. Focal radioligand uptake above the background that could not be explained by physiological uptake was assessed as suspicious of malignancy. Foci that demonstrated low uptake on early scans and no uptake on late imaging without any anatomical features suggesting malignancy (e.g., size, shape, contrast enhancement) were not regarded as PCa associated and excluded from the further analysis. All prostate cancer-related findings were categorized according to the PROMISE criteria including miTNM expression score (0 = no expression; 1 = low expression; 2 = intermediate expression; 3 = high expression) and staging (miTr = local recurrence; miN1 = single pelvic lymph node region with lymph node metastases; miN2 = lymph node metastases in > 1 pelvic lymph node region; miM1a = extrapelvic lymph node metastasis; miM1b = bone metastasis; miM1c = metastasis to other site) [25]. To improve the readability of the [^68^ Ga]Ga-PSMA-I&T PET/CT staging results in the text and the illustrations, the prefix “mi” was left out from here on. Separate miTNM stagings were specified for early and late scans. Lesions outside the pelvis, seen only on early whole-body imaging, were included in both stagings. A final miTNM staging was derived from the joint information of early and late scans combined. All changes of the miTNM were regarded as relevant if no distant metastases were present (< Tx Nx M1).

Semiquantitative image analysis was conducted for up to five pelvic lesions that were regarded as characteristic for PCa. The SUVmax, SUVmean, and SUVpeak were calculated from regions of interest drawn around each lesion with isocontours set at 40% of the maximum. The SUVmean of the background was measured in the gluteus muscle. The SUVmean of the blood-pool activity was calculated from regions of interest drawn in the distal abdominal aorta. If the aorta was not included in the scan volume of the pelvis on late imaging, the common iliac artery was used. In these individual cases, care was taken not to include the vessel wall into the measurement by correlating the placement of the region of interest with the contrast-enhanced high-resolution CT. The SUVmean of the urine was measured in the bladder. All measurements were conducted using the imaging processing package Fiji for Image Processing and Analysis in Java, Version 1.53c [26, 27].

### Follow-up information

Clinical follow-up information was regarded as viable if [^68^ Ga]Ga-PSMA-I&T PET/CT, PSA values, or histological results were available after localized therapy (i.e., radiotherapy of the prostatic fossa/pelvic lymphatics or salvage lymph node dissection; *n* = 26, 8.8%) or no treatment (i.e., watchful waiting; *n* = 16, 5.4%). If a systemic therapy (e.g., androgen deprivation) had been initiated, no validation of the lesions with pathological radioligand uptake on [^68^ Ga]Ga-PSMA-I&T PET/CT was possible (*n* = 72, 24.2%). No adequate follow-up was accessible in 183 (61.6%) patients.

### Statistical analysis

Continuous variables are described with median and IQR. Differences between continuous variables were evaluated with the Wilcoxon signed-rank test (paired samples) and the Mann–Whitney U test (unpaired samples). Binary logistic regression analysis was used to investigate potential influence factors regarding the change of case interpretation due to additional late imaging of the pelvis. All *p* values of < 0.05 were considered statistically significant. Statistical analyses were conducted with STATA® version 17.0 (STATA Corp, College Station, Texas, USA). A Sankey diagram was created using SankeyMATIC [28].

## Results

Of 297 PSMA PET/CT scans, 134 (45.1%) displayed PSMA-positive lesions suggestive of recurrent prostate cancer. Early scans were positive in 114 (38.4%) cases. Additional late imaging of the pelvis showed lesions characteristic for PCa in 131 (44.1%) men. Scan results differed depending on the PSA value at the time of imaging and the scanner (Table 2). The employed PET/CT system did not have an influence on changes to the case interpretation due to biphasic imaging (odds ratio 1.37, 95% confidence interval 0.78–2.42, *p* = 0.27).

**Table 2:** PET/CT case positivity rate by PSA level and scanner

PSA (ng/ml)	PET/CT positive (*n* = 297) (%)	Gemini GXL 10 positive (*n* = 136) (%)	Vereos positive (*n* = 161) (%)
< 0.2	28.9	20.0	36.0
0.2–0.5	38.5	24.6	50.6
> 0.5–1	53.7	38.9	65.2
> 1	66.7	44.8	85.3
All	45.1	30.1	57.8

*PSA* prostate-specific antigen value at the time of PET/CT

The SUVmean of the background (early median 0.4, IQR 0.3–0.4 vs. late median 0.3, IQR 0.2–0.3) and the blood-pool (early median 1.1, IQR 1.0–1.4 vs. late median 0.7, 0.6–0.9) decreased from early to late imaging (*p* < 0.01, respectively). The SUVmean of the urine increased between the two time points (early median 15.0, IQR 9.4–24.2 vs. late median 19.1, IQR 12.0–34.4; *p* < 0.01).

### Lesion analysis

**Fig. 2:**
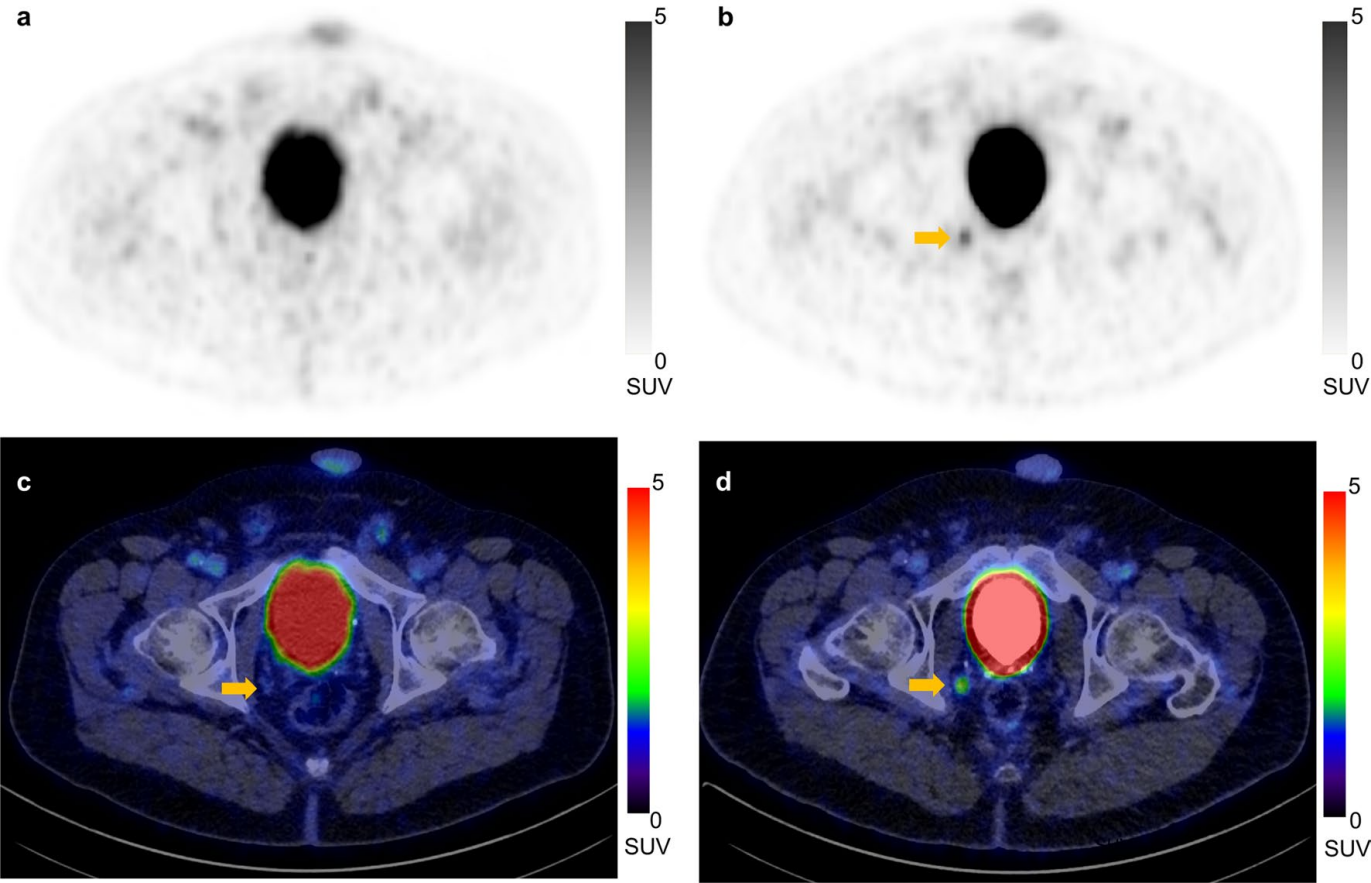
PET (**a**, **b**) and PET/CT fusion **c**, **d** images of the pelvis with increasing uptake of [.^68^ Ga]Ga-PSMA-I&T of a lymph node (arrows) in the deep internal iliac lymph node region on the right side (early scans: a and c; late scans: b and d)

One hundred and ninety-five PCa characteristic lesions were analyzed in the cohort. One hundred and forty-four (73.8%) lesions were detected correctly on early images (Tr: 38, N: 85, M1b: 21). Seventeen foci (10.6%; Tr: 1, N: 16, M1b: 0) with low uptake on early scans, no uptake on late imaging, and no anatomical features suggesting malignancy were excluded from the final consensus. One hundred and ninety-one (97.9%) of the analyzed lesions were visible on late scans (Tr: 60, N: 108, M1b: 23) including 51 that were not perceivable on early imaging (Tr: 24, N: 25, M1b: 2). Figure 2 shows a representative PET/CT of a pelvic lymph node metastasis that only demonstrated relevant tracer uptake on late imaging. Four (2.1%) lesions regarded as characteristic for PCa were only detectable on early scans (Tr: 2, N: 2, M1b: 0).

**Fig. 3:**
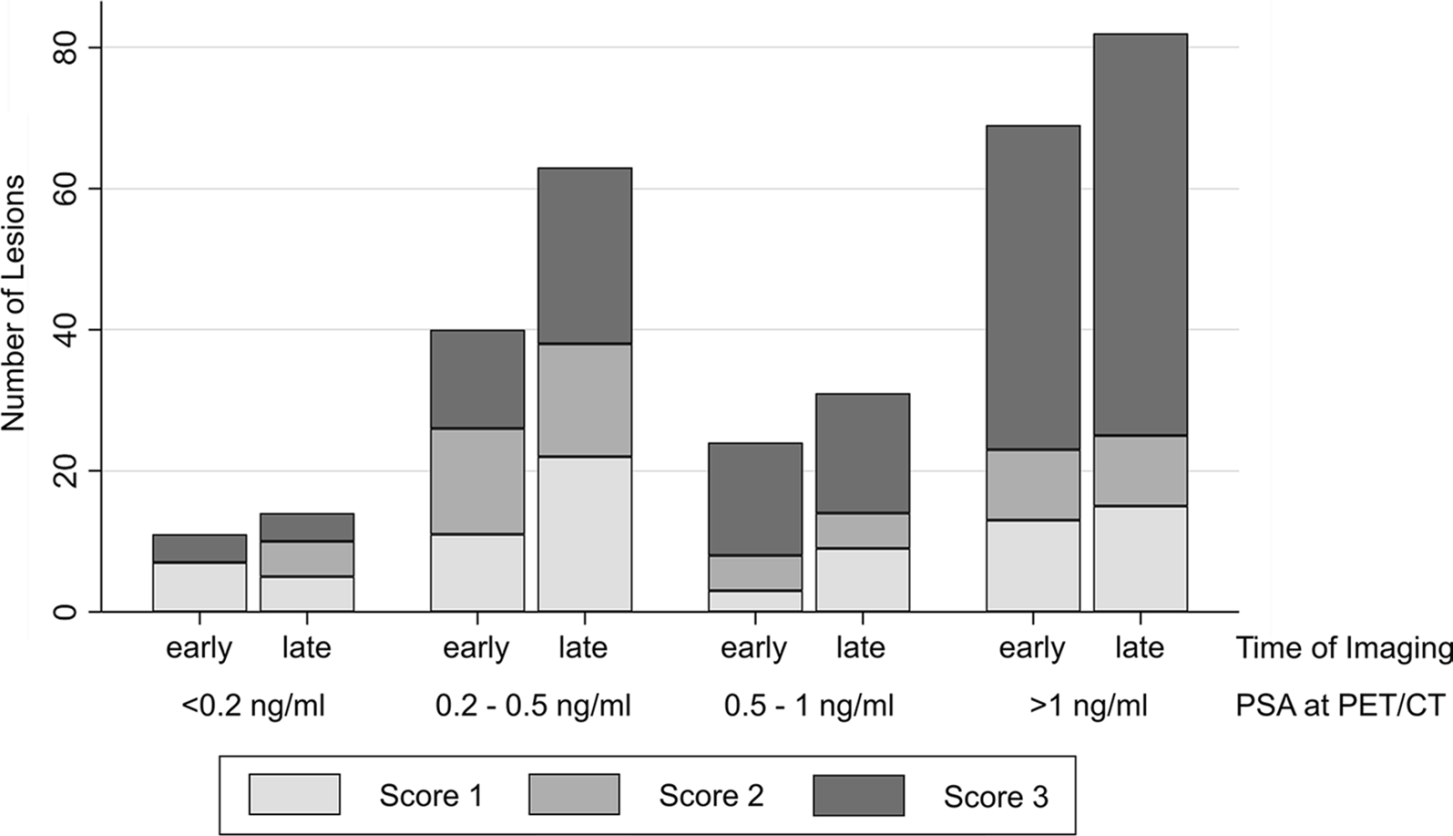
PSMA expression score according to PROMISE criteria [25] and number of PCa-characteristic lesions identified on early and late imaging with the corresponding prostate-specific antigen (PSA) values at the time of PET/CT

The miTNM expression score of lesions at the different imaging time points is depicted in Fig. 3. Scores of local recurrences (early median 1, IQR 0–3 vs. late median 3, IQR 2–3) and lymph node lesions of the pelvis (early median 2, IQR 1–3 vs. late median 3, IQR 1–3) increased significantly (*p* < 0.01, respectively). Scores of bone lesions did not change (early median 2, IQR 2–3 vs. late median 3, IQR 1–3; *p* = 0.2).

A similar development was seen regarding the lesions’ SUV. In locally recurrent tumors and lymph node metastases, the SUVmax was significantly higher on late imaging than on early scans (*p* < 0.01, respectively) but did not change in bone metastases (*p* = 0.1). Due to a more pronounced decrease in the background, the tumor-to-background ratio of all three categories increased significantly between the imaging time points (Table 3). The SUVpeak of local recurrences (early median 2.3, IQR 0.4–4.0 vs. late median 3, IQR 2.3–4.7) and lymph nodes (early median 2.2, IQR 1.3–4.2 vs. late median 2.8, IQR 1.7–5.7) increased significantly from early to late scans (*p* < 0.01, respectively). However, the SUVpeak of bone metastases did not change (early median 2.6, IQR 2.1–4.9 vs. late median 3.0, IQR 2.0–5.6; *p* = 0.1).

**Table 3:** SUVmax and SUVmax to background values

miTNM	Early imaging median (IQR)	Late imaging median (IQR)	*p *value
Tr	3.0 (0.4–5.7)	3.9 (3.2–5.8)	< 0.01
Tr to BG	9.1 (1.2–15.4)	14.4 (10.2–29.0)	< 0.01
N	3.4 (1.4–6.9)	4.0 (2.3–9.4)	< 0.01
N to BG	9.9 (4.3–19.5)	16.0 (8.4–35.0)	< 0.01
M1b	3.5 (2.8–7.0)	4.7 (2.8–8.7)	0.1
M1b to BG	9.3 (6.4–21.6)	18.1 (11.1–37.2)	< 0.01
All	3.4 (0.4–6.5)	3.9 (2.6–8.2)	< 0.01
All to BG	9.4 (1.7–19.1)	15.5 (9.6–34.1)	< 0.01

*BG* background; *IQR* interquartile range

Foci that were downstaged showed a lower SUVmax (median 1.7, IQR 1.5–2.2; *p* = 0.01) and SUVpeak (median 1.5, IQR 1.2–1.8; *p* = 0.02) than lesions that were included in the final miTNM staging.

### Changes of case interpretation and miTNM staging

Additional late imaging of the pelvis led to changes between the interpretation of early PET/CT scans alone and the final evaluation in 63 (21.2%) patients. Four (1.3%) patients showed lesions that were only positive on early imaging (Table 4, Additional file 1: Table S1 and Additional file 1: Table S2). None of the investigated clinical factors demonstrated a statistically significant association with the change of case interpretation due to additional late imaging of the pelvis (Table 5).

**Table 4:** Cases with changes of lesion interpretation in biphasic imaging of the pelvis

PSA (ng/ml)	Upstaging on late imaging	Downstaging on late imaging	Up- and downstaging on late imaging	Lesion only visible on early imaging	No differences
< 0.2	3 (6.7%)	2 (4.4%)	0	0	40 (88.9%)
0.2–0.5	23 (15.5%)	10 (6.8%)	2 (1.4%)	3 (2.0%)	110 (74.3%)
0.5–1	7 (17.1%)	1 (2.4%)	0	1 (2.4%)	32 (78.0%)
> 1	13 (20.6%)	1 (1.6%)	1 (1.6%)	0	48 (76.2%)
All	46 (15.5%)	14 (4.7%)	3 (1.0%)	4 (1.3%)	230 (77.4%)

Values in parentheses are percentages of row totals

*PSA* prostate-specific antigen value at the time of PET/CT

**Table 5:** Association of clinical variables and change in case interpretation due to additional late imaging of the pelvis

Variable	OR	95% CI	*p* value
PSA at RP	0.97	0.93–1.01	0.19
*pT*			
pT2	Reference category		
pT3	1.07	0.61–1.88	0.81
pT4	1	–	–
*ISUP grade*			
1	Reference category		
2	1.11	0.23–5.44	0.89
3	1.14	0.23–5.69	0.87
4	1.29	0.14–11.54	0.82
5	2.25	0.4–12.67	0.36
Resection margin positive	0.79	0.33–1.89	0.59
Nodal status positive	0.89	0.42–1.91	0.77
PSA at PET/CT	0.91	0.76–1.08	0.26

*CI* confidence interval; *ISUP* international society of urological pathology; *OR* odds ratio; *pT* pathological T-stage; *PSA* prostate-specific antigen; and *RP* radical prostatectomy

**Fig. 4:**
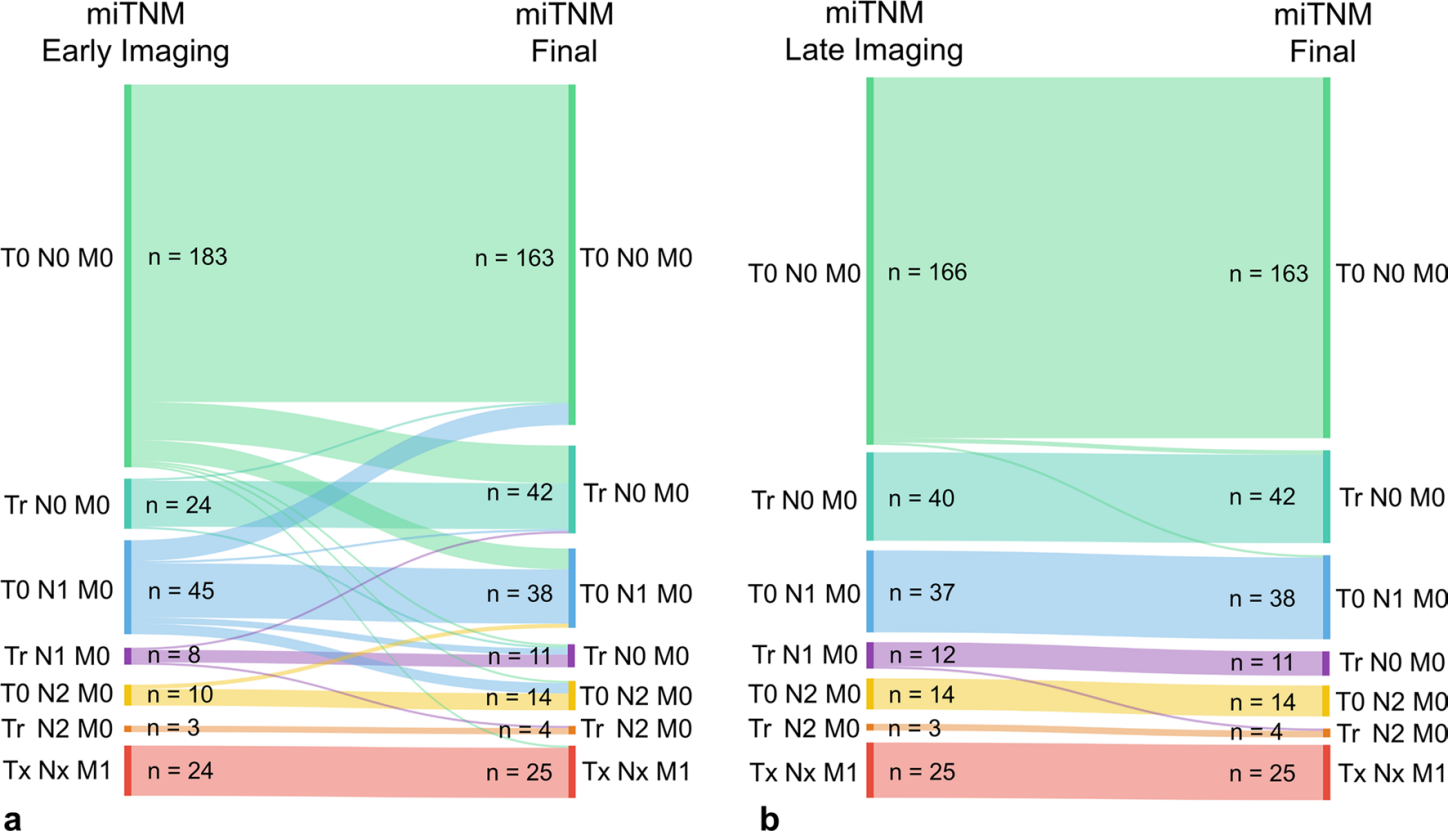
Sankey diagrams of the miTNM staging differences of early [68 Ga]Ga-PSMA-I&T PET/CT versus the final miTNM staging (**a**) and of late [68 Ga]Ga-PSMA-I&T PSMA PET/CT versus the final miTNM staging (**b**). The number of changes is depicted by the strength of lines connecting the respective miTNM stagings

The resulting differences of the miTNM between early imaging vs. the final case evaluation and late scans vs. the final miTNM are summarized in Fig. 4. Changes to the miTNM of early scans compared to the final evaluation were seen in 58 (19.5%) cases. Thirty-one (10.4%) patients who were initially evaluated as T0 N0 M0 on early whole-body imaging were upstaged because of the added information of additional late scans of the pelvis (Tr: 19; Nx: 12, M1b: 1) (Additional file 1: Table S3). In 27 (9.1%) cases, late imaging led to up- and/or downstaging of patients with a positive early PET/CT result (> T0 N0 M0). This included 5 men whose T-stage differed (T0 → Tr: 4; Tr → T0: 1) and 21 cases with differences in their nodal status (N0 → Nx: 1; Nx → N0: 12; N1 ↔ N2: 8). Moreover, two patients with evidence of distant metastases were upstaged due to late scans (T0 N0 M1b → Tr N0 M1b and T0 N0 M1b → T0 N1 M1b).

Three patients with multiple positive pelvic lymph nodes in more than one lymph node region on early scans (N2) showed additional positive lymph nodes of the pelvis on late imaging. Two cases demonstrated simultaneous up- and downstaging of pelvic lymph nodes between early and late PET leaving the miTNM unchanged.

Lastly, 4 (2.1%) lesions in 4 (1.3%) patients were only identified on early images leading to an upstaging of the final miTNM compared to late scans (Additional file 1: Table S4).

### Histopathological findings and follow-up information

Sixteen (5.4%) patients received no treatment and demonstrated increasing PSA values in all but one case (PSA stable at 0.3 ng/ml for 1 year). In 11 of these patients, follow-up PSMA PET/CT was available (6 with no change, 5 with progression).

Eleven (3.7%) patients underwent radiotherapy of the prostatic fossa (T0 N0 M0 or Tr N0 M0: 8) or radiation of the prostatic fossa and the pelvic lymphatics (T0 N2 M0: 3). The PSA values decreased below 0.2 ng/ml in all of these cases after treatment.

On 15 (5.1%) patients, salvage lymphadenectomy was performed. Lymph node metastases were found in the locations described on prior PSMA PET/CT in 13 patients. In 1 of these cases, 1 PSMA-positive lesion could not be identified during surgery. The PSA value, however, decreased below 0.1 ng/ml after surgery indicating that this focus was probably false positive on imaging. Additional lymph node metastases that had not been PSMA-positive on prior PET/CT were identified in 3 patients. In 2 cases, no metastases were found during salvage surgery. PSA values increased in these patients, and pathological lymph nodes were identified on follow-up imaging (PET/CT or contrast-enhanced CT) at the same sites as on the initial PSMA PET/CT scans.

## Discussion

Additional late scans of the pelvis in [^68^ Ga]Ga-PSMA-I&T PET/CT showed more lesions and improved contrast compared to early imaging. This led to substantial changes of the final miTNM staging in recurrent prostate cancer of patients who had only undergone radical prostatectomy.

Prospective trials have already shown that PSMA PET/CT has a significant impact on treatment decisions in patients with biochemical recurrence [8–13] which, among others, has led to its recommendation in this scenario [6]. Despite the central role of PSMA PET/CT in PCa, imaging protocols still vary between institutions potentially influencing patient care. The overall detection rate per patient of 45.1% in the presented cohort is lower than in other studies which investigated biphasic imaging with ^68^ Ga-labeled radioligands [17, 19, 21–24]. Possible reasons for this are the low initial tumor grade and considerably lower PSA value at the time of imaging in this cohort. Further on, patients were imaged using two different PET/CT scanners, an analogue and a digital system. Due to an inferior sensitivity and signal-to-noise ratio, analogue scanners were reported to have lower detection rates than digital PET/CT [29] reducing the overall number of positive scans in the presented cohort. Moreover, the analysis was conducted in a highly selected patient group after prostatectomy without pre- or postoperative treatments like radiation or androgen deprivation therapy. Comparable to other publications [17, 21, 23, 24], the lesion-to-background ratio increased for local recurrences, lymph node, and bone metastases. Also, the gain in the case detection rate on late imaging of 5.7% falls into the range of 0–10% reported in other publications [18, 19, 21, 22, 24]. However, the relationship between imaging of a patient at two time points is often more complex than the comparison of positive vs. negative cases. Individual lesions of the same patient may demonstrate divergent uptake kinetics as reported by Afshar et al. [19]. This can lead to simultaneous upstagings and downstagings. Further on, the clinical relevance of changes varies depending on the localization of the recurrent disease. Newly detected lesions outside the field of standard salvage therapy or signs of systemic disease have a large impact on case assessment. On the other hand, an additional metastasis in an otherwise already systemic disease spread seems less important. To address the relevance of these changes as good as possible, all scans were evaluated using the PROMISE criteria and miTNM classification proposed by Eiber et al. [25]. To our knowledge, no other study that investigated biphasic imaging used this approach to date. Unfortunately, most publications did not distinguish between pelvic and extrapelvic lymph node metastases [17, 19, 21–24]. Hoffman et al. only found two additional extrapelvic lymph node metastases and one additional bone metastasis on late abdominal imaging in a total of 554 lesions in 114 positive scans of 178 men. The additional lesions were seen in patients who already had other PCa-related manifestations [18]. In the presented cohort, 51 new lesions of the pelvis were detected on late scans only and 17 foci that were regarded as suspicious on early images were downstaged in the final consensus. Most changes to the lesion interpretation occurred at a PSA of 0.2–0.5 ng/ml (Table 4). The increasing SUVmax values of most lesions and the higher tumor-to-background ratio on late scans are two important reasons for the upstagings on late imaging, especially of lesions with a low PSMA expression. Changes of the miTNM occurred in about 20% of patients. Differences between the results of Hoffmann et al. and the presented data may have arisen from different imaging protocols, the lower injected dose, and substantially lower PSA at imaging in our patient group. The same reasons can be responsible for the only minor impact of additional late scans described by Schmuck et al. who also used [^68^ Ga]Ga-PSMA-I&T for imaging of patients with biochemical recurrence after prostatectomy or radiation therapy [24].

The upstagings from T0 N0 M0 in the investigated cohort included 18 cases with signs of local recurrences on late scans alone (Tr N0 M0). While this does not necessarily change the decision to conduct salvage therapy or influence the standard field of radiotherapy, it may be seen as a prognostic factor. Emmett et al. reported that the freedom from disease progression was significantly longer in these patients compared to men with pelvic nodal or distant metastases [30]. All other upstagings from T0 N0 M0 to positive lymph nodes of the pelvis can direct salvage radiotherapy (e.g., focal boost on the metastasis) or salvage lymph node dissection with/without PSMA-radioguidance [31, 32]. The additional detection of a bone metastasis without other PSMA-positive PCa-related lesions occurred in only one case. Nevertheless, it demonstrates potential weaknesses of monophasic imaging. Upstagings or downstagings in cases with lesions suggestive for PCa-recurrence already on early PSMA PET/CT (i.e., > T0 N0 M0) occurred in about half of all patients with staging differences due to additional late scans. Comparable to upstagings from T0 N0 M0, these changes may improve the accuracy of the assessment of the disease prognosis and direct clinical decisions. Staging differences in patients with distant metastases on early imaging were only seen in two cases. These are usually of minor clinical significance reducing the impact of additional late scans in this patient subgroup. Lesions that were exclusively positive on early scans occurred only in about 1% of cases. Even so, the results of the current analysis do not support monophasic late imaging of patients with early PSA failure after prostatectomy. The uptake dynamic, especially of local recurrences and pelvic lymph node metastases, is seen as an important factor in the lesion interpretation. Moreover, none of the investigated clinical factors showed a significant association with the change of case interpretation due to additional late scans of the pelvis. Consequently, biphasic imaging of the pelvis in [^68^ Ga]Ga-PSMA-I&T PET/CT of patients with early PSA relapse after radical prostatectomy should be considered in clinical routine.

Some limitations of the presented study should be acknowledged. Patients did not receive diuretics before imaging. The use of furosemide prior to PET acquisition was reported to potentially improve detectability of PCa-related lesions by lowering the urine activity [33, 34]. The increasing urine activity between the imaging time points in the presented study is a drawback of the implemented protocol which might have masked lesions close to the ureter or bladder. However, 96% of patients received a contrast-enhanced CT demarcating the urinary tract on late imaging. This approach can help to identify ureter artifacts and mitigate the influence of the renal excretion of [^68^ Ga]Ga-PSMA-I&T on image interpretation. Second, clinical routine led to a relatively wide range of the early uptake time possibly influencing case interpretation. Further on, almost half of all scans were conducted with an analogue system that demonstrated lower detection rates on early and late imaging than the digital PET/CT scanner. The presented results may, therefore, underestimate the influence of additional late imaging of the pelvis on newer scanners. Particularly, the number of upstagings due to late imaging may be higher than in the investigated group (Additional file 1: Table S1 and Additional file 1: Table S2). However, a substantial number of changes in the case interpretation were seen in both systems implicating the potential for improvement of detection rates with biphasic imaging of the pelvis regardless of the scanner. Next, additional late scans were restricted to the pelvis potentially missing extrapelvic metastases that would only have been visible on late imaging. Although the risk seems to be very low in the investigated cohort, distant metastases are possible even in low-risk cases [35]. To reduce the chance of missing relevant visceral or bone metastases, contrast-enhanced full-dose CT scans were performed whenever possible. Moreover, these images facilitated the differentiation of lymph nodes and ganglia, a common pitfall in PSMA PET/CT [34]. The restriction of the scan volume to the pelvis on late imaging made it necessary to calculate SUVmean values of the blood-pool from regions of interest drawn into the common iliac artery if the aorta was not included. Accidental inclusion of the vessel wall due to the smaller diameter of the iliac arteries may have influenced the observed results. However, to reduce the chance of bias, the placement of the regions of interest was correlated with the contrast-enhanced CT scans if possible. Unfortunately, follow-up information was not available in most cases. Nevertheless, the data of patients that were treated at our institution after [^68^ Ga]Ga-PSMA-I&T PET/CT indicated that the applied protocol led to adequate lesion detection. Lastly, the impact of PSMA PET/CT on clinical outcomes like progression-free survival is promising [32, 36]. Imaging protocols that improve lesion detection rates may thus have a positive impact on treatment planning and outcomes. But further investigations are needed to evaluate this topic as well as the influence of PSMA-targeting imaging on PCa-related mortality which is still unknown.

## Conclusions

Additional late scans of the pelvis in [^68^ Ga]Ga-PSMA-I&T PET/CT of men with PSA recurrence after radical prostatectomy without other prior treatments had a substantial impact on lesion detectability and miTNM staging. No clinical predictors were significantly associated with these findings. Routine additional late imaging of the pelvis should be considered in this particular patient group. However, further analyses are needed to evaluate the influence of biphasic imaging on clinical endpoints.

## Supplementary Information

**Additional file1**.** Table S1**. Gemini GXL 10: Cases with changes of lesion interpretation in biphasic imaging of the pelvis. PSA = prostate-specific antigen value in ng/ml at the time of PET/CT. Values in parentheses are percentages of row totals.** Table S2**. Vereos: Cases with changes of lesion interpretation in biphasic imaging of the pelvis. PSA = prostate-specific antigen value in ng/ml at the time of PET/CT. Values in parentheses are percentages of row totals.** Table S3**. Differences between final miTNM and miTNM of early images.** Table S4**. Differences between final miTNM and miTNM of late images.

## Data Availability

The datasets generated during and/or analyzed during the current study are available from the corresponding author on reasonable request.

## Abbreviations

IQR: Interquartile range
PCa: Prostate cancer
PSA: Prostate-specific antigen
PSMA: Prostate-specific membrane antigen

## References

[1] Data explorer | ECIS [Internet]. [cited 2022 Aug 28]. Available from: https://ecis.jrc.ec.europa.eu/

[2] Pompe RS, Gild P, Karakiewicz PI, Bock LP, Schlomm T, Steuber T (2018). Long-term cancer control outcomes in patients with biochemical recurrence and the impact of time from radical prostatectomy to biochemical recurrence. Prostate.

[3] Freedland SJ, Humphreys EB, Mangold LA, Eisenberger M, Dorey FJ, Walsh PC (2007). Death in patients with recurrent prostate cancer after radical prostatectomy: prostate-specific antigen doubling time subgroups and their associated contributions to all-cause mortality. J Clin Oncol.

[4] Roehl KA, Han M, Ramos CG, Antenor JA, Catalona WJ (2004). Cancer progression and survival rates following anatomical radical retropubic prostatectomy in 3,478 consecutive patients: long-term results. J Urol.

[5] Van den Broeck T, van den Bergh RCN, Arfi N, Gross T, Moris L, Briers E (2019). Prognostic value of biochemical recurrence following treatment with curative intent for prostate cancer: a systematic review. Eur Urol.

[6] EAU Guidelines. Edn. presented at the EAU Annual Congress Amsterdam 2022. [Internet]. Arnhem, The Netherlands; [cited 2022 May 15]. Available from: https://uroweb.org/guideline/prostate-cancer/

[7] Lowrance WT, Breau RH, Chou R, Chapin BF, Crispino T, Dreicer R (2021). Advanced prostate cancer: AUA/ASTRO/SUO guideline part I. J Urol.

[8] Fendler WP, Ferdinandus J, Czernin J, Eiber M, Flavell RR, Behr SC (2020). Impact of 68Ga-PSMA-11 PET on the management of recurrent prostate cancer in a prospective single-arm clinical trial. J Nucl Med.

[9] Calais J, Fendler WP, Eiber M, Gartmann J, Chu FI, Nickols NG (2018). Impact of 68 Ga-PSMA-11 PET/CT on the management of prostate cancer patients with biochemical recurrence. J Nucl Med.

[10] Hope TA, Aggarwal R, Chee B, Tao D, Greene KL, Cooperberg MR (2017). Impact of 68Ga-PSMA-11 PET on management in patients with biochemically recurrent prostate cancer. J Nucl Med.

[11] Roach PJ, Francis R, Emmett L, Hsiao E, Kneebone A, Hruby G (2018). The impact of 68 Ga-PSMA PET/CT on management intent in prostate cancer: results of an australian prospective multicenter study. J Nucl Med.

[12] Cerci JJ, Fanti S, Lobato EE, Kunikowska J, Alonso O, Medina S (2022). Diagnostic performance and clinical impact of 68Ga-PSMA-11 PET/CT imaging in early relapsed prostate cancer after radical therapy: a prospective multicenter study (IAEA-PSMA Study). J Nucl Med.

[13] Ceci F, Rovera G, Iorio GC, Guarneri A, Chiofalo V, Passera R (2022). Event-free survival after 68 Ga-PSMA-11 PET/CT in recurrent hormone-sensitive prostate cancer (HSPC) patients eligible for salvage therapy. Eur J Nucl Med Mol Imaging.

[14] Fendler WP, Eiber M, Beheshti M, Bomanji J, Ceci F, Cho S (2017). 68Ga-PSMA PET/CT: joint EANM and SNMMI procedure guideline for prostate cancer imaging: version 1.0. Eur J Nucl Med Mol Imaging.

[15] Strauss DS, Sachpekidis C, Kopka K, Pan L, Haberkorn U, Dimitrakopoulou-Strauss A (2021). Pharmacokinetic studies of [68 Ga]Ga-PSMA-11 in patients with biochemical recurrence of prostate cancer: detection, differences in temporal distribution and kinetic modelling by tissue type. Eur J Nucl Med Mol Imaging.

[16] Uprimny C, Kroiss AS, Decristoforo C, Fritz J, Warwitz B, Scarpa L (2017). Early dynamic imaging in 68Ga- PSMA-11 PET/CT allows discrimination of urinary bladder activity and prostate cancer lesions. Eur J Nucl Med Mol Imaging.

[17] Sahlmann CO, Meller B, Bouter C, Ritter CO, Ströbel P, Lotz J (2016). Biphasic 68Ga-PSMA-HBED-CC-PET/CT in patients with recurrent and high-risk prostate carcinoma. Eur J Nucl Med Mol Imaging.

[18] Hoffmann MA, Buchholz HG, Wieler HJ, Rosar F, Miederer M, Fischer N (2020). Dual-time point [68ga]ga-psma-11 pet/ct hybrid imaging for staging and restaging of prostate cancer. Cancers (Basel).

[19] Afshar-Oromieh A, Sattler LP, Mier W, Hadaschik BA, Debus J, Holland-Letz T (2017). The clinical impact of additional late PET/CT imaging with 68Ga-PSMA-11 (HBED-CC) in the diagnosis of prostate cancer. J Nucl Med.

[20] Alberts I, Sachpekidis C, Gourni E, Boxler S, Gross T, Thalmann G (2020). Dynamic patterns of [68Ga]Ga-PSMA-11 uptake in recurrent prostate cancer lesions. Eur J Nucl Med Mol Imaging.

[21] Hohberg M, Kobe C, Täger P, Hammes J, Schmidt M, Dietlein F (2019). Combined early and late [68Ga]PSMA-HBED-CC PET scans improve lesion detectability in biochemical recurrence of prostate cancer with low PSA levels. Mol Imaging Biol.

[22] Morawitz J, Kirchner J, Hertelendy J, Loberg C, Schimmöller L, Dabir M (2022). Is there a diagnostic benefit of late-phase abdomino-pelvic PET/CT after urination as part of whole-body 68 Ga-PSMA-11 PET/CT for restaging patients with biochemical recurrence of prostate cancer after radical prostatectomy?. EJNMMI Res.

[23] Derlin T, Schmuck S, Juhl C, Zörgiebel J, Schneefeld SM, Walte ACA (2018). PSA-stratified detection rates for [68Ga]THP-PSMA, a novel probe for rapid kit-based 68Ga-labeling and PET imaging, in patients with biochemical recurrence after primary therapy for prostate cancer. Eur J Nucl Med Mol Imaging.

[24] Schmuck S, Nordlohne S, von Klot CA, Henkenberens C, Sohns JM, Christiansen H (2017). Comparison of standard and delayed imaging to improve the detection rate of [68Ga]PSMA I&T PET/CT in patients with biochemical recurrence or prostate-specific antigen persistence after primary therapy for prostate cancer. Eur J Nucl Med Mol Imaging.

[25] Eiber M, Herrmann K, Calais J, Hadaschik B, Giesel FL, Hartenbach M (2018). Prostate cancer molecular imaging standardized evaluation (PROMISE): proposed miTNM Classification for the interpretation of PSMA-Ligand PET/CT. J Nucl Med.

[26] Schindelin J, Arganda-Carreras I, Frise E, Kaynig V, Longair M, Pietzsch T (2012). Fiji: an open-source platform for biological-image analysis. Nat Methods.

[27] Free and Open source PET/CT Viewer [Internet]. [cited 2022 Jul 12]. Available from: https://www.researchgate.net/project/Free-and-Open-source-PET-CT-Viewer

[28] SankeyMATIC: A Sankey diagram builder for everyone [Internet]. [cited 2022 May 22]. Available from: https://sankeymatic.com/

[29] Alberts I, Prenosil G, Sachpekidis C, Weitzel T, Shi K, Rominger A (2020). Digital versus analogue PET in [68Ga]Ga-PSMA-11 PET/CT for recurrent prostate cancer: a matched-pair comparison. Eur J Nucl Med Mol Imaging.

[30] Emmett L, Tang R, Nandurkar R, Hruby G, Roach P, Watts JA (2020). 3-year freedom from progression after 68Ga-PSMA PET/CT-Triaged management in men with biochemical recurrence after radical prostatectomy: results of a prospective multicenter trial. J Nucl Med.

[31] Knipper S, Mehdi Irai M, Rauscher I, Simon R, Eiber M, van Leeuwen FW (2022). Cohort study of oligorecurrent prostate cancer patients: Oncological outcomes of patients treated with salvage lymph node dissection via PSMA radioguided surgery. Eur Urol.

[32] Schmidt-Hegemann NS, Stief C, Kim TH, Eze C, Kirste S, Strouthos I (2019). Outcome after PSMA PET/CT–based salvage radiotherapy in patients with biochemical recurrence after radical prostatectomy: a 2-institution retrospective analysis. J Nucl Med.

[33] Derlin T, Weiberg D, von Klot C, Wester HJ, Henkenberens C, Ross TL (2016). 68Ga-PSMA I&T PET/CT for assessment of prostate cancer: evaluation of image quality after forced diuresis and delayed imaging. Eur Radiol.

[34] Alberts I, Niklas-Hünermund J, Sachpekidis C, Zacho HD, Mingels C, Dijkstra L (2021). Combination of forced diuresis with additional late imaging in 68Ga-PSMA-11 PET/CT: effects on lesion visibility and radiotracer uptake. J Nucl Med.

[35] Ferdinandus J, Fendler WP, Farolfi A, Washington S, Mohammad O, Pampaloni MH (2021). PSMA PET validates higher rates of metastatic disease for European Association of Urology Biochemical Recurrence Risk Groups: an international multicenter study. J Nucl Med.

[36] Rogowski P, Trapp C, von Bestenbostel R, Eze C, Ganswindt U, Li M (2022). Outcome after PSMA-PET/CT-based salvage radiotherapy for nodal recurrence after radical prostatectomy. Eur J Nucl Med Mol Imaging.

