# Supplementary material for: Evaluation of [68 Ga]Ga-PSMA-I&T PET/CT with additional late scans of the pelvis in prostate-specific antigen recurrence using the PROMISE criteria
Source: EJNMMI Res. 2022 Oct 9;12:66. doi: 10.1186/s13550-022-00938-3 (PMC9548463; doi:10.1186/s13550-022-00938-3)
Supplement: Supplementary file 1 — Additional file1. Table S1. Gemini GXL 10: Cases with changes of lesion interpretation in biphasic imaging of the pelvis. PSA = prostate-specific antigen value in ng/ml at the time of PET/CT. Values in parentheses are percentages of row totals. Table S2. Vereos: Cases with changes of lesion interpretation in biphasic imaging of the pelvis. PSA = prostate-specific antigen value in ng/ml at the time of PET/CT. Values in parentheses are percentages of row totals. Table S3. Differences between final miTNM and miTNM of early images. Table S4. Differences between final miTNM and miTNM of late images. [file 13550_2022_938_MOESM1_ESM.docx]

**Supplementary Material**

| **Supplementary Table 1** Gemini GXL 10: Cases with changes of lesion interpretation in biphasic imaging of the pelvis | | | | | |
| --- | --- | --- | --- | --- | --- |
|  | Upstaging on late imaging | Downstaging on late imaging | Up- and downstaging on late imaging | Lesion only visible on early imaging | No differences |
| <0.2 | 0 | 2 (10.0%) | 0 | 0 | 18 (90.0%) |
| 0.2 – 0.5 | 7 (10.1%) | 6 (8.7%) | 1 (1.4%) | 1 (1.4%) | 54 (78.3%) |
| 0.5 – 1 | 2 (11.1%) | 1 (5.6%) | 0 | 1 (5.6%) | 14 (77.8%) |
| >1 | 5 (17.2%) | 1 (5.6%) | 0 | 0 | 23 (79.3%) |
| All | 14 (10.3%) | 10 (7.4%) | 1 (0.7%) | 2 (1.5%) | 109 (80.1%) |
| PSA = prostate-specific antigen value in ng/ml at the time of PET/CT  Values in parentheses are percentages of row totals. | | | | | |

| **Supplementary Table 2** Vereos: Cases with changes of lesion interpretation in biphasic imaging of the pelvis | | | | | |
| --- | --- | --- | --- | --- | --- |
|  | Upstaging on late imaging | Downstaging on late imaging | Up- and downstaging on late imaging | Lesion only visible on early imaging | No differences |
| <0.2 | 3 (12.0%) | 0 | 0 | 0 | 22 (88.0%) |
| 0.2 – 0.5 | 16 (20.3%) | 4 (5.1%) | 1 (1.3%) | 2 (2.5%) | 56 (70.9%) |
| 0.5 – 1 | 5 (21.7%) | 0 | 0 | 0 | 18 (78.3%) |
| >1 | 8 (23.5%) | 0 | 1 (2.9%) | 0 | 25 (73.5%) |
| All | 32 (19.9%) | 4 (2.5%) | 2 (1.2%) | 2 (1.2%) | 121 (75.2%) |
| PSA = prostate-specific antigen value in ng/ml at the time of PET/CT  Values in parentheses are percentages of row totals. | | | | | |

|  | **Supplementary Table 3** Differences between final miTNM and miTNM of early images | | | | | | | |
| --- | --- | --- | --- | --- | --- | --- | --- | --- |
|  | **Final miTNM** | | | | | | | |
| **miTNM early imaging** |  | T0 N0 M0 | Tr N0 M0 | T0 N1 M0 | Tr N1 M0 | T0 N2 M0 | Tr N2 M0 | Tx Nx M1 |
|  | T0 N0 M0 | 152 | 18 | 10 | 1 | 1 | 0 | 1 |
|  | Tr N0 M0 | 1 | 22 | 0 | 1 | 0 | 0 | 0 |
|  | T0 N1 M0 | 10 | 1 | 26 | 3 | 5 | 0 | 0 |
|  | Tr N1 M0 | 0 | 1 | 0 | 6 | 0 | 1 | 0 |
|  | T0 N2 M0 | 0 | 0 | 2 | 0 | 8 | 0 | 0 |
|  | Tr N2 M0 | 0 | 0 | 0 | 0 | 0 | 3 | 0 |
|  | Tx Nx M1 | 0 | 0 | 0 | 0 | 0 | 0 | 24 |
|  |  |  |  |  |  |  |  |  |

|  | **Supplementary Table 4** Differences between final miTNM and miTNM of late images | | | | | | | |
| --- | --- | --- | --- | --- | --- | --- | --- | --- |
|  | **Final miTNM** | | | | | | | |
| **miTNM late imaging** |  | T0 N0 M0 | Tr N0 M0 | T0 N1 M0 | Tr N1 M0 | T0 N2 M0 | Tr N2 M0 | Tx Nx M1 |
|  | T0 N0 M0 | 163 | 2 | 1 | 0 | 0 | 0 | 0 |
|  | Tr N0 M0 | 0 | 40 | 0 | 0 | 0 | 0 | 0 |
|  | T0 N1 M0 | 0 | 0 | 37 | 0 | 0 | 0 | 0 |
|  | Tr N1 M0 | 0 | 0 | 0 | 11 | 0 | 1 | 0 |
|  | T0 N2 M0 | 0 | 0 | 0 | 0 | 14 | 0 | 0 |
|  | Tr N2 M0 | 0 | 0 | 0 | 0 | 0 | 3 | 0 |
|  | Tx Nx M1 | 0 | 0 | 0 | 0 | 0 | 0 | 25 |
|  |  |  |  |  |  |  |  |  |
